# Quality of Life in Women with Gestational Diabetes Mellitus: A Systematic Review

**DOI:** 10.1155/2017/7058082

**Published:** 2017-02-23

**Authors:** Daniela Marchetti, Danilo Carrozzino, Federica Fraticelli, Mario Fulcheri, Ester Vitacolonna

**Affiliations:** ^1^Department of Psychological Health and Territorial Sciences, “G. d'Annunzio” University of Chieti-Pescara, Chieti, Italy; ^2^Psychiatric Research Unit, Psychiatric Centre North Zealand, Copenhagen University Hospital, Hillerød, Denmark; ^3^Department of Medicine and Aging, “G. d'Annunzio” University of Chieti-Pescara, Chieti, Italy

## Abstract

*Background and Objective*. Diagnosis of Gestational Diabetes Mellitus (GDM) could significantly increase the likelihood of health problems concerning both potential risks for the mother, fetus, and child's development and negative effects on maternal mental health above all in terms of a diminished Quality of Life (QoL). The current systematic review study is aimed at further contributing to an advancement of knowledge about the clinical link between GDM and QoL.* Methods*. According to PRISMA guidelines, PubMed, Web of Science, Scopus, and Cochrane databases were searched for studies aimed at evaluating and/or improving levels of QoL in women diagnosed with GDM.* Results*. Fifteen research studies were identified and qualitatively analyzed by summarizing results according to the following two topics: GDM and QoL and interventions on QoL in patients with GDM. Studies showed that, in women with GDM, QoL is significantly worse in both the short term and long term. However, improvements on QoL can be achieved through different intervention programs by enhancing positive diabetes-related self-management behaviors.* Conclusion*. Future studies are strongly recommended to further examine the impact of integrative programs, including telemedicine and educational interventions, on QoL of GDM patients by promoting their illness acceptance and healthy lifestyle behaviors.

## 1. Introduction

Gestational Diabetes Mellitus (GDM) is defined as “diabetes diagnosed in the second or third trimester of pregnancy that was not clearly overt diabetes prior to gestation” [1]. GDM is one of the most frequent metabolic diseases during pregnancy and approximately affects 7% (range: 2–18%) of all pregnancies [2–5]. This clinical condition potentially affects not only negative medical outcomes but also the mental health status with additional adverse consequences on psychological well-being and Quality of Life (QoL) [6, 7].

Pregnancy is a particular time for all women. This condition becomes even more delicate when there is a diagnosis of GDM which makes necessary controls and therapies that will inevitably affect the woman's life. GDM can lead to potential risks for the mother, fetus, and child's development, as well as clinically relevant negative effects on maternal mental health, above all in terms of a diminished QoL [8, 9].

Health-related QoL was extensively accepted as a highly relevant outcome in different clinical trials [10]. QoL potentially operates as a unifying concept that comprises many domains such as general, physical, and psychological health, positive social relationships, environmental mastery, purpose in life, self-acceptance, autonomy, and personal growth factors [3, 11]. However, it may act by a core mechanism of subjective appraisal of own health status [12] resulting in specific diagnostic and therapeutic implications [13]. That is, QoL dimension can explain the different individual response to a standard medical treatment leading to an incomplete recovery in terms of health perception [14].

This concept was further highlighted by the World Health Organization criteria [15], which stressed the clinical relevance to promote the health status by not only treating physical symptoms but also instilling a positive mental state [16]. In this regard, the current systematic review study is aimed at further contributing to an advancement of knowledge about the clinical link between GDM and QoL.

## 2. Materials and Methods

### 2.1. Information Sources and Searches

According to Preferred Reporting Items for Systematic Reviews and Meta-Analyses (PRISMA) guidelines [17], a comprehensive electronic search strategy was used to identify peer-reviewed articles assessing QoL experienced by pregnant women diagnosed with GDM up to 19 October 2016. The following keywords were used: “gestational diabetes OR gestational hyperglycemia OR hyperglycemic pregnancy” combined using AND Boolean operator with “quality of life OR well-being”. After the initial search was performed, studies were screened for eligibility; their relevance was initially assessed using titles and abstracts and finally the full review of papers. Searching and eligibility of target responses were carried out independently by two investigators (DM and DC); any type of disagreements was resolved by consensus among these primary raters and a senior investigator (EV).

Electronic research-literature databases searched included PubMed, Web of Science, Scopus, and Cochrane databases. In order to detect any missed articles during the literature search, reference lists of candidate articles were reviewed for further studies not yet identified. For each excluded study, we determined which elements of the electronic search were not addressed.

### 2.2. Eligibility Criteria

Papers were eligible for inclusion if they were research reports in English language describing data on QoL domains in relation to GDM diagnosis during pregnancy. We focused on studies examining QoL in women with* GDM* directly or evaluating the link between QoL and well-being alternatively by specifically using measures testing QoL. Based on this inclusion criterion, we selected studies referring to QoL or well-being related to QoL by consequently excluding research reports assessing well-being through measures on negative mental health (i.e., depression, anxiety, bipolar mood, and distress) or by focusing on a medical definition of well-being (health status, wellness, physical health, etc.). Also studies aimed at evaluating the effectiveness of programs targeted for GDM patients providing effects on QoL were included.

We excluded peer-reviewed single-case studies, reviews, meta-analyses, letters to the editor and commentaries, conference abstracts, books, and papers that were clearly irrelevant. In order to generate conclusions specific to GDM, we excluded research reports aimed at addressing levels of QoL in diabetic patients, without reporting specific data for GDM subgroup (e.g., considering diabetic pregnant women those with preexisting diabetes and those receiving the first diagnosis of GDM during pregnancy). No limit was set with regard to publication date.

### 2.3. Analysis and Data Synthesis

The heterogeneous nature of the identified studies (in terms of design and measures) did not permit a formal meta-analysis. Hence, narrative synthesis approach was judged to be the most appropriate method for the review.

Studies were categorized based on the object of the study, differentiated as those that aimed at assessing association and those that evaluated interventions. Significant information for each study was summarized and compared.

## 3. Results

**Figure 1:**
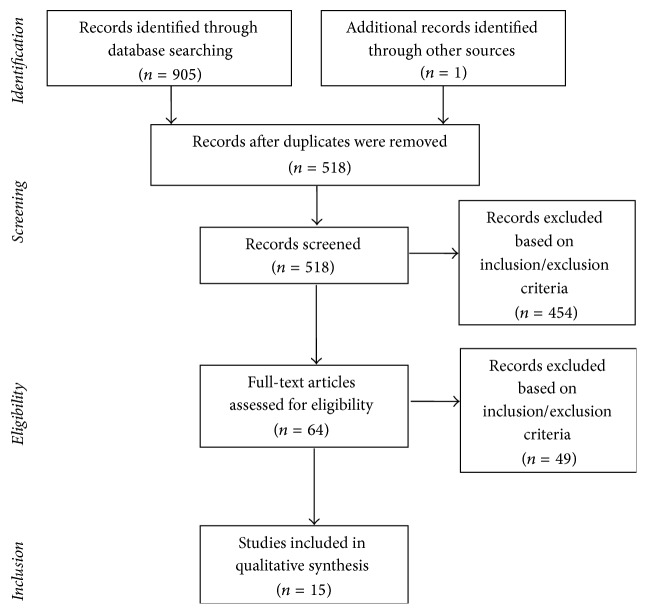
Flowchart of the systematic search.

The search of PubMed, Web of Science, Scopus, and Cochrane databases, including additional manual search, initially provided a total of 906 articles, as shown in the PRISMA flowchart (see Figure 1). Fifteen research studies were identified as clearly relevant and qualitatively analyzed in the systematic review. Pertinent results were summarized according to the following two specific chapters: (a) GDM and QoL (Table 1) and (b) interventions on QoL in patients with GDM (Table 2). Criteria for the diagnosis of GDM used in reviewed studies are illustrated in Table 3. In all but two studies [9, 26], standardized measures of QoL were used (see Tables 1 and 2 for details).

### 3.1. GDM and QoL

When combining an evaluation of levels of illness acceptance with an assessment of the QoL, Bień et al. [3] have found that illness acceptance was significantly correlated with all QoL related scores (*p* < 0.05) with *R* values ranging from 0.20 to 0.54. Similarly, GDM participants who did not report an individual illness interference with everyday life obtained significantly higher scores compared to those stating an illness limitation on specific QoL factors. That is, statistical differences between groups were observed on general QoL (*p* = 0.006), perceived general health (*p* = 0.006), and physical (*p* = 0.003), psychological (*p* = 0.01), and environmental (*p* = 0.0001) domains. Moreover, further examining QoL dimensions, the psychological domain was slightly worse than other QoL domains [3].

A similar study from Kopec et al. [19] evidenced that 171 respondents (i.e., 87.3% of the total sample specifically used for statistical analyses) reported a negative impact of GDM on their social life. Results also showed that higher levels of distress were significantly reported by women who tested glucose levels more frequently and those under treatment with insulin. In addition, patients perceiving insufficient information about their GDM symptoms reported higher levels of distress than those perceiving adequate information [19].

Another recent study from Danyliv et al. [18] compared levels of health-related QoL (HRQoL) of GDM patients with those of women with normal glucose tolerance (NGT) during pregnancy. They found significantly lower scores on HRQoL dimension for the group of GDM patients. However, when adjusting for the effects of other clinical covariates by performing a pooled multivariate analysis, the authors [18] showed that GDM per se did not influence HRQOL levels.

Similarly, a study of Dalfrà et al. [8] compared levels of QoL between GDM patients, pregnant women with type 1 diabetes, and healthy pregnant participants. GDM respondents scored significantly lower than healthy controls on the SF-36 general health perception subscale (*p* < 0.05) as evaluated during pregnancy at the third trimester. Moreover, whereas the SF-36 domains of QoL significantly improved after delivery among all three groups, rates of the SF-36 general health perception subscale remained significantly lower in GDM patients than in healthy controls [8].

Results in contrast with aforementioned findings were obtained from a previous research study aimed at evaluating impact of GDM on HRQOL after delivery. The authors [21] revealed no statistically significant differences between GDM patients and the healthy control group on the HRQOL dimensions. Similar results were previously reported also by Mautner et al. [22]. Their study underlined no clinically relevant differences between women with GDM and the healthy control group as concerns HRQoL levels.

Different results were later obtained from Trutnovsky et al. [20] who demonstrated significantly diminished levels of QoL among GDM patients. Specifically, GDM participants showed, from mid to late pregnancy, a significant reduction of physical, psychological, social, and global scores on the World Health Organization Quality of Life subscales (WHOQOL-BREF).

Results in line with above reported data were addressed by Rumbold and Crowther [24] when evidencing that women positively screened for GDM showed lower health perceptions than those with negative screening (*p* < 0.05). However, these statistical differences between positive and negative screened women for GDM were not significant late in pregnancy [24].

Similar results were provided in a study from Kim et al. [23]. This research highlighted that women with a diagnosis of GDM were significantly more prone to report poor physical function and a worse self-rated health status compared to healthy pregnant women. The same result was further supported after delivery with a greater proportion of GDM pregnant women reporting a lower self-rated health status than healthy controls. In addition, self-rated health status further worsened in the third trimester among women with GDM compared to healthy pregnant women without GDM. Surprisingly, GDM clinical condition was not significantly associated with declines in any other SF-36 health status subscales in the third trimester [23].

Finally, Lapolla et al. [9] indirectly revealed a worsening level of QoL among GDM women when demonstrating that this diagnosis resulted in the development of anxiety symptoms.

### 3.2. Interventions on QoL in Patients with GDM

When comparing QoL levels in GDM women attending different treatment programs (i.e., metformin alone, insulin alone, or a combination of both treatments), Latif et al. [25] showed that the negative influence on overall QoL was less with metformin compared to insulin. Nevertheless, the treatment combining metformin with insulin resulted in a greater negative impact on the QoL. Concerning the evaluation of treatment outcomes, Elnour et al. [28] observed statistically significant (*p* < 0.05) improvements in the HRQoL among patients attending a pharmacological care program.

A study, testing the effect of a telemedicine intervention, by Dalfrà and colleagues [27] found a significant improvement of the SF-36 general health perception, energy/vitality, and mental health subscales among participants attending the intervention group. Similar results were later obtained from Petkova et al. [26]. They found a significant improvement in QoL of women with GDM involved in an intervention program aimed at educating about diet, exercise, self-monitoring, and insulin treatment.

Finally, Crowther et al. [29] randomly assigned women with GDM to a specific intervention group aimed to provide dietary advice, blood glucose monitoring, and insulin therapy. At three months after delivery, participants reported a significant improvement in their QoL by obtaining higher scores on the SF-36.

## 4. Discussion

QoL is a clinically relevant concept determining the individual evaluation of own health status. This subjective appraisal seems to act, above all physical and treatment components of diabetes during pregnancy, as a psychological factor affecting medical outcomes in GDM. Based on current research studies, we have found that QoL could be significantly compromised, both short term and long term, when women cope with pregnancy complicated by GDM. However, GDM per se does not seem to act as unique clinical variable negatively affecting different levels of QoL among women with GDM. That is, the relationship between GDM medical symptoms and QoL domains could be mediated by a complex interaction of several factors whose unifying psychological element can be identified through the concept of the illness experience [30]. The potential underlying psychological factor, operating as core variable to clinically explain the different QoL status among patients with GDM, may consist of the varying individual way to respond to own bodily symptoms. Such psychological component conceived as general health perception was originally investigated by Mechanic and Volkart [31]. In this regard, they provided a definition of “the ways in which symptoms may be differentially perceived, evaluated, and acted upon by different kinds of persons” [30]. A relatively recent review study of Lawrence [32] has further underlined how perceptions and expectations of women with GDM may significantly affect their psychological and behavioral response during and after pregnancy.

To the very best of our knowledge, this is the first review study systematically analyzing the impact of GDM and its symptoms on QoL levels. Indeed, only a previous recently published systematic review study, evaluating the health status and QoL in postpartum women, was fulfilled by Van Der Woude et al. [33]. However, the authors neglected the assessment of such psychological mechanisms in women with GDM.

Based on additional relevant results from a review study examining the influence of QoL in the treatment outcomes of diabetes [34], specific implications can be identified. The medical evaluation of GDM should comprise a clinically valid psychological assessment of QoL in order to attempt monitoring its potential effects on GDM prognosis from first diagnosis, during the treatment, and after delivery. Specifically, the gold standard should comprise self-rating scales as screening measures for identifying psychological comorbidities potentially leading to adverse and negative clinical consequences during diabetes [35].

As regards the intervention studies examining the impact of specific treatments on QoL dimensions among women with GDM, promising results were found. The five studies retrieved [25–29] clearly indicate the efficacy of different therapeutic programs to improve QoL by enhancing positive diabetes self-management behaviors such as balanced diet, exercise, self-monitoring, and insulin control. Despite this, much more studies are largely needed when taking into account heterogeneity in subjects and study design as well as the evidence that the control group and experimental group were not consistent across studies we have qualitatively examined.

Further research should be conducted to test the effect of integrative programs on QoL focusing on pharmacological care mixed up with advanced practices based on information and communication technologies (e.g., telemedicine and/or games for health) [36, 37]. In this regard, the major aim is to educate patients on healthier lifestyle habits (healthy diet and physical activity domains) [38, 39] and to facilitate the process of illness acceptance [40] after a diagnosis of GDM.

Finally, our review shows some noteworthy points: (1) the positive effect of a telemedicine intervention on both diabetes-related medical outcomes and general health perception, energy/vitality, and mental health; (2) a significant improvement in QoL of women with GDM attending an educational program. In the future, these considerations should be taken into account for the management of diabetes during pregnancy.

## Figures and Tables

**Table 1:** Characteristics of included studies assessing the impact of GDM on Quality of Life.

Study	Country/setting/year	Study design	Aim	*N*	Measure of Quality of Life
Bień et al. [3]	Poland Three hospitals 2016	Observational study without control group	To assess the factors affecting QoL (and illness acceptance) in pregnant women diagnosed with GDM.	114 pregnant women with GDM	WHOQOL-BREF

Danyliv et al. [18]	Ireland Five antenatal centers involved in The Atlantic Diabetes in Pregnancy (DIP) initiative 2015	Observational study with control group	To compare QoL between GDM and NGT women 2 to 5 years after pregnancy. To explore participants characteristics which may influence their QoL.	234 women diagnosed with GDM during pregnancy 108 women with a history of healthy pregnancy	Visual Analog Scale of the EQ-5D-3L

Kopec et al. [19]	Poland University-affiliated GDM clinic 2015	Observational study without control group (longitudinal)	To assess, among other objectives, factors affecting the QoL of pregnant women with GDM.	205 pregnant women diagnosed with GDM	SF-8

Trutnovsky et al. [20]	Austria University GDM clinic 2012	Observational study without control group (prospective)	To evaluate, among other outcomes, QoL of women treated for GDM.	45 pregnant women affected by GDM, divided based on type of treatment: 27 diet-treated women 18 diet plus insulin women	WHOQOL-BREF

Lapolla et al. [9]	Italy Ten diabetes centers specialized in the care of pregnant women 2012	Observational study without control group	To evaluate QoL, wishes, and needs of Italian and immigrant women diagnosed with GDM.	286 pregnant women affected by GDM, divided into two groups: 198 Italian women 88 immigrant women	QoL is only indirectly evaluated with questions covering feelings and concerns related to the diagnosis of GDM and its treatment

Dalfrà et al. [8]	Italy Twelve diabetes clinics 2012	Observational study with control group	To examine the impact of diabetes on QoL among pregnant women with diabetes (GDM and T1DM) compared with pregnant women with a normal glucose tolerance.	245 women divided into three groups: 176 pregnant women with GDM 30 pregnant women with T1DM 39 healthy controls (pregnant women with negative GCT or OGTT findings)	SF-36

Halkoaho et al. [21]	Finland University hospital 2010	Observational study with control group	To evaluate the effects of GDM on women's QoL after delivery.	77 women diagnosed with GDM during pregnancy 54 healthy controls (women with normal glucose tolerance tests during pregnancy)	15D HRQoL

Mautner et al. [22]	Austria Public hospital 2009	Observational study with control group (longitudinal)	To explore QoL (and the incidence of depressive symptoms) in women during pregnancy and after delivery, comparing women with GDM, hypertensive disorders, and risk for preterm delivery with a control group characterized by uncomplicated pregnancy.	90 pregnant women, divided into four groups: 18 women affected by hypertensive disorders 11 women affected by GDM 32 women at risk of preterm delivery 29 healthy controls (pregnancy without complications)	WHOQOL-BREF

Kim et al. [23]	California, USA Six hospitals 2005	Observational study with control group (prospective)	To investigate the influence of GDM (and PIH) on maternal health status, testing the hypothesis that, among others, women with GDM would have a great likelihood to report declines in health status than women without GDM, with at least a partial mediation by cesarean birth or preterm delivery.	1445 pregnant women divided into three groups: 64 women with GDM 148 women with PIH 1233 healthy women	Physical functioning scale, vitality scale, and self-rated health item of the SF-36

Rumbold and Crowther [24]	South Australia Hospital with a high-risk pregnancy service and neonatal intensive care unit 2002	Observational study with control group (prospective)	To survey pregnant women on their experience of being screened for GDM, testing the hypothesis that women with a GDM diagnosis would experience a reduction in QoL (perception of the pregnancy, their health, and that of their baby) compared with women with a negative screening result.	145 pregnant women divided into two groups 21 with positive OGTT (GDM group) 124 with negative OGTT	SF-36

GDM: Gestational Diabetes Mellitus; NGT: normal glucose tolerance; OGTT: oral glucose tolerance test; QoL: Quality of Life; PIH: pregnancy-induced hypertension; T1DM: type 1 diabetes mellitus; EQ-5D-3L: 3-level version of the EuroQol 5-Dimension; 15D HRQoL: 15-Dimensional Health-Related Quality of Life; SF-36: 36-Item Short-Form Health Survey; SF-8: 8-Item Short-Form Health Survey; WHOQOL-BREF: World Health Organization Quality of Life-BREF.

**Table 2:** Characteristics of included studies assessing the impact of treatments on Quality of Life among GDM pregnant women.

Study	Country/setting/year	Study design	Aim	*N*	Measure of Quality of Life
Latif et al. [25]	England Two hospitals 2013	Quasi-experimental study Three conditions: (1) Treatment with metformin (2) Treatment with insulin (3) Treatment with metformin + insulin	To examine QoL (and treatment satisfaction) of women affected by GDM receiving metformin alone, insulin alone, or a combination of both treatments.	128 women diagnosed with GDM during pregnancy assigned to one of three conditions: 68 treated with metformin 32 treated with insulin 28 treated with metformin + insulin	ADDQoL

Petkova et al. [26]	Bulgaria Antenatal clinic 2011	Quasi-experimental study Two conditions: (1) Education group (intervention) (2) Control group	To investigate the effectiveness of an educational program for pregnant women with GDM on their QoL (and other outcomes).	30 pregnant women affected by GDM assigned to one of two conditions: 15 assigned to the intervention condition (education program) 15 assigned to the control condition	Five items measuring QoL

Dalfrà et al. [27]	Italy Twelve diabetes clinics 2009	Quasi-experimental study Two conditions: (1) Telemedicine condition (intervention) (2) Usual care condition (control)	To assess the effect of a telemedicine intervention on QoL (and other outcomes) among pregnant women with diabetes (GDM and T1DM).	235 pregnant women divided into two groups: 203 women with GDM 32 women with T1DM Participants of each group were sequentially assigned to one of two conditions: 88 women affected by GDM and 17 women affected by T1DM assigned to the telemedicine condition 115 women affected by GDM and 15 women affected by T1DM assigned to the control condition	SF-36

Elnour et al. [28]	United Arab Emirates Hospital 2008	Experimental study (RCT) Two conditions: (1) Pharmaceutical care program (intervention) (2) Usual care (control)	To evaluate the effect of a pharmaceutical care intervention programme for women with GDM on QoL (and other outcomes) both during pregnancy and after delivery.	180 pregnant women diagnosed with GDM randomly assigned to one of two conditions: 108 assigned to the intervention condition 72 assigned to the control condition	SF-36

Crowther et al. [29]	New South Wales; Queensland; South Australia; England; Wales Eighteen antenatal clinics 2005	Experimental study (RCT) Two conditions: (1) Intervention group (2) Routine-care group (control)	To examine the effect of an intervention (including dietary advice, blood glucose monitoring, and insulin therapy) for women with GDM on QoL (and other primary and secondary outcomes).	1000 pregnant women with GDM, randomly assigned to one of two conditions: 490 assigned to the intervention condition 510 assigned to the control condition	SF-36

GDM: Gestational Diabetes Mellitus; QoL: Quality of Life; T1DM: type 1 diabetes mellitus; ADDQoL: Audit of Diabetes-Dependent Quality of Life; SF-36: 36-Item Short-Form Health Survey.

**Table 3:** Diagnostic criteria for GDM of included studies.

Study	Time and methods of diagnosis	Diagnostic criteria
Bień et al. [3]	Diabetes first diagnosed during pregnancy, in accordance with the current guidelines of the Polish Diabetology Society: for pregnant women with risk factors, the 75 g OGTT is required. If glycemia is normal, the test should be readministered at 24–28 weeks of pregnancy or when first symptoms indicative of diabetes are observed. For women without risk factors, the 75 g OGTT is administered at 24–28 weeks of pregnancy	75-g OGTT: FPG levels of 92–125 mg/dl and/or 1 h PG level ≥180 mg/dl and/or 2 h PG level of 153–199 mg/dl

Danyliv et al. [18]	Pregnant women were offered screening at 24–28 weeks' gestation using a 75 g OGTT	75 g OGTT in accordance with the IADPSG criteria

Kopec et al. [19]	GDM was diagnosed by a two-step approach. The oral GCT administered between 24 and 28 weeks of pregnancy. Women with 1 h PG level >180 mg/dl (10.0 mmol/l) in the OGCT were classified as having GDM. Women with PG levels between 140 mg/dl (7.8 mmol/l) and 180 mg/dl (10.0 mmol/l) were referred for a diagnostic 75 g OGTT	Two-step approach: 50 g GCT followed, if positive, by 75 g OGTT interpreting the results according to the WHO diagnostic criteria

Latif et al. [25]	GDM was diagnosed at 28 weeks of pregnancy	A 2 h 75 g OGTT according to the WHO diagnostic criteria

Trutnovsky et al. [20]	Diagnosed GDM based on the results of an elevated 75 g OGTT	Diagnostic criteria not fully specified

Lapolla et al. [9]	GDM was diagnosed according to Carpenter and Coustan's criteria	Carpenter and Coustan's criteria

Dalfrà et al. [8]	Screening for GDM was done with a GCT between the 24th and 28th weeks of gestation, and the diagnosis was confirmed with a 100 g OGTT, interpreting the results according to the Recommendations of the 4th International Workshop Conference on GDM	Carpenter and Coustan's criteria

Petkova et al. [26]	Women, suspected to have GDM, were subjected to 2 h 75 g GCT. Those with sugar level around 140 mg/dl (7.8 mmol/L) or above were requested for OGTT recommended by WHO	Two-step approach: GCT + OGTT interpreting the results according to the WHO diagnostic criteria

Halkoaho et al. [21]	The diagnosis of GDM is based on a 2 h glucose tolerance test generally administered during the 24th–28th weeks of pregnancy to women with GDM risk factors	Diagnostic criteria not fully specified

Mautner et al. [22]	The group “gestational diabetes” included women diagnosed with a pathological oral glucose tolerance test requiring insulin therapy at the end of the second and the beginning of the third trimester. The diagnosis of GDM is based on a pathological OGTT	Diagnostic criteria not fully specified

Dalfrà et al. [27]	Screening for GDM was done with a GCT between the 24th and 28th weeks of gestation, and the diagnosis was confirmed with a 100 OGTT	Carpenter and Coustan's criteria

Elnour et al. [28]	Women within the first 20 weeks of gestation confirmed diagnosis of GDM	Diagnostic criteria not specified

Kim et al. [23]	GDM was diagnosed according to Carpenter and Coustan's criteria between 24–28 weeks of gestation	Carpenter and Coustan's criteria

Crowther et al. [29]	Pregnancy between 16 and 30 weeks' gestation with one or more risk factors for gestational diabetes on selective screening or a positive 50 g GCT and a 75 g OGTT at 24 to 34 weeks' gestation in accordance with the WHO diagnostic criteria	Two-step or one-step approach: 50 g GCT: 1 h PG level ≥140 mg/dl (7.8 mmol/l) + 75 g OGTT: 2 h PG level of 140–198 (7.8–11.0 mmol/l) or selective screening: 75 g OGTT interpreting the results according to the WHO diagnostic criteria

Rumbold and Crowther [24]	Universal antenatal screening for GDM by either a random blood sample or a 50 g GCT at 24 to 28 weeks' gestation. Women who screen positive are offered a diagnostic 75 g OGTT	Two-step approach: random blood sample or 50 g GCT followed, if positive, by 75 g OGTT interpreting the results according to the WHO diagnostic criteria

GCT: glucose challenge test; GDM: Gestational Diabetes Mellitus; IADPSG: International Association of Diabetes and Pregnancy Study Groups; OGTT: oral glucose tolerance test; WHO: World Health Organization.
